# Anti-Interleukin-1 Beta/Tumor Necrosis Factor-Alpha IgY Antibodies Reduce Pathological Allergic Responses in Guinea Pigs with Allergic Rhinitis

**DOI:** 10.1155/2016/3128182

**Published:** 2016-03-07

**Authors:** Hu Wei-xu, Zhou Wen-yun, Zhu Xi-ling, Wen Zhu, Wu Li-hua, Wu Xiao-mu, Wei Hui-ping, Wang Wen-ding, He Dan, Xiang Qin, Hu Guo-zhu

**Affiliations:** ^1^Department of Radiation Oncology, Shanghai Proton and Heavy Ion Center, Shanghai 201315, China; ^2^Institute of Clinical Medical Sciences, Jiangxi Province People's Hospital, Nanchang, Jiangxi 330006, China; ^3^Department of Hematology, Jiangxi Academy of Medical Science, Nanchang, Jiangxi 330006, China; ^4^State Key Laboratory of Chemo/Biosensing and Chemometrics, College of Biology, Hunan University, Changsha, Hunan 410082, China

## Abstract

This study aims to determine whether the combined blockade of IL-1*β* and TNF-*α* can alleviate the pathological allergic inflammatory reaction in the nasal mucosa and lung tissues in allergic rhinitis (AR) guinea pigs. Healthy guinea pigs treated with saline were used as the healthy controls. The AR guinea pigs were randomly divided into (1) the AR model group treated with intranasal saline; (2) the 0.1% nonspecific IgY treatment group; (3) the 0.1% anti-TNF-*α* IgY treatment group; (4) the 0.1% anti-IL-1*β* IgY treatment group; (5) the 0.1% combined anti-IL-1*β* and TNF-*α* IgY treatment group; and (6) the fluticasone propionate treatment group. The inflammatory cells were evaluated using Wright's staining. Histopathology was examined using hematoxylin-eosin staining. The results showed that the number of eosinophils was significantly decreased in the peripheral blood, nasal lavage fluid, and bronchoalveolar lavage fluid (*P* < 0.05), and eosinophil, neutrophil, and lymphocyte infiltration and edema were significantly reduced or absent in the nasal mucosa and lung tissues (*P* < 0.05) in the combined 0.1% anti-IL-1*β*- and TNF-*α* IgY-treated guinea pigs. The data suggest that topical blockade of IL-1*β* and TNF-*α* could reduce pathological allergic inflammation in the nasal mucosa and lung tissues in AR guinea pigs.

## 1. Introduction

Allergic rhinitis (AR) is an IgE-mediated type I hypersensitivity inflammatory disease of the nasal mucosa. IgE bound to Fc*ε*RI on mast cells and eosinophils is cross-linked by allergens, resulting in the release of diverse preformed and newly synthesized mediators to promote the local recruitment and activation of leukocytes and the production of inflammatory cytokines and T helper 2 (Th2) cytokines, which contribute to the development of late-phase reactions (2 h to 24 h after exposure to an allergen). Our previous study demonstrated that proinflammatory cytokines (IL-1*β*, TNF-*α*, IL-18, IL-22, and IL-33), Th2 cytokines (IL-5, IL-9, and IL-13), TGF-*β*_1_, and OVA-specific IgE levels in the peripheral blood (PB) and nasal lavage fluid (NLF) were significantly decreased by an intranasal instillation of 0.1% combined anti-IL-1*β* and anti-TNF-*α* IgY antibodies in ovalbumin- (OVA-) induced AR guinea pigs [1]. Eosinophil infiltration in the nasal mucosa was increased in AR guinea pigs [2] and mice [3]. The total number of inflammatory cells, primarily eosinophils, in the bronchoalveolar lavage fluid (BALF) and pulmonary tissues was increased in OVA-sensitized guinea pigs [4] and rats [5]. In addition, the pathogenesis of allergic rhinitis is linked to asthma [6]. Inhibition of proinflammatory cytokines is effective for controlling and alleviating allergic inflammation because proinflammatory cytokines precede Th2 cytokines in the pathological response [4].

In the present study, we aim to determine whether the combined blockade of IL-1*β* and TNF-*α* can alleviate pathological allergic inflammatory reactions and reduce inflammatory cell infiltration in the nasal mucosa and lung tissues in OVA-induced AR guinea pigs. These results demonstrate that combined anti-IL-1*β* and TNF-*α* IgY antibodies block IL-1*β* and TNF-*α* inflammatory cytokines and that this action is a mechanism for the treatment of allergic rhinitis. Our study provided strong experimental evidence that supports a novel therapeutic strategy against AR.

## 2. Material and Methods

### 2.1. Animals

Hartley guinea pigs (male, 7 weeks old, 230 g ± 40 g) were purchased from the National Center for Experimental Animal Seed Rodent Shanghai Sub-Centres (Production license SXCK (Hu) 2012-0008, Shanghai, China). The experimental studies in guinea pigs were performed in accordance with the animal experiment guidelines established by the Ministry of Science and Technology of the People's Republic of China. The animal procedures have been approved by the Jiangxi Province People's Hospital Ethics Committee. The room where the experiments were performed was free of noise and strong odors, had a controlled temperature of 23 ± 2°C and 60 ± 5% relative humidity, and had a 12-hour light and 12-hour dark cycle. The guinea pigs had free access to water and food.

### 2.2. Establishment of a Guinea Pig Model of Allergic Rhinitis and the Experimental Groups

After adaptation for 7 days, the guinea pigs were divided into a healthy control group (group C) (*n* = 17), in which the guinea pigs were sensitized on days 1, 3, 5, 7, 9, 11, and 13 using a 1.0 mL intraperitoneal injection of 0.9% saline, and challenged from days 21–30 by instilling the nostrils with 0.2 mL of 0.9% saline (0.1 mL/each nostril), and the AR groups. The sensitization and challenge protocol described by Bahekar et al. [7] and Guo-Zhu et al. [1] was used in the AR groups. In the procedure for systemic sensitization, the guinea pigs were sensitized on days 1, 3, 5, 7, 9, 11, and 13 using a 1.0 mL intraperitoneal injection of OVA (300 *μ*g/animal) (Grade II, Sigma, USA) and aluminum hydroxide (30 mg/animal) (Thermo-Fisher Scientific, USA) in 0.9% saline in the AR groups. The sensitization success rate was 95.65% (132/138 animals) via OVA intracutaneous testing [7, 8]. For the topical challenge procedure at 7 days after the last systemic sensitization, the guinea pigs were challenged from days 21–30 by instilling the nostrils with 0.2 mL of an OVA solution (2.0 mg/0.1 mL/each nostril) in the AR groups. After three OVA challenges, guinea pigs exhibiting AR were randomly divided into six groups based on their allergic symptom scores (the guinea pigs in each group included strong, mild, and weak allergic reactions). (1) The AR model group (group M) (*n* = 15) was treated with 0.9% saline and an OVA solution for seven days by instilling the nostrils with 0.2 mL of OVA solution after instilling the nostrils with 0.2 mL of 0.9% saline (0.1 mL/each nostril). (2) The 0.1% nonspecific IgY treatment group (group Z_1_) (*n* = 18) was treated with 0.1% nonspecific IgY (prepared in the laboratory, purity 85%, and valence combined recombinant human IL-1*β* and TNF-*α*: 1 : 8/mg protein) [1] and an OVA solution for seven days by instilling the nostrils with 0.2 mL of OVA solution after instilling the nostrils with 0.2 mL of 0.1% nonspecific IgY (0.1 mL/each nostril). (3) The 0.1% anti-TNF-*α* IgY treatment group (group Z_2_) (*n* = 17) was treated with 0.1% anti-TNF-*α* IgY (prepared in the laboratory, purity 85%, and valence combined recombinant human TNF-*α*: 1 : 3200/mg protein) [1] and an OVA solution for seven days by instilling the nostrils with a 0.2 mL of an OVA solution after instilling the nostrils with 0.2 mL of 0.1% anti-TNF-*α* IgY (0.1 mL/each nostril). (4) The 0.1% anti-IL-1*β* IgY treatment group (group Z_3_) (*n* = 17) was treated with 0.1% anti-IL-1*β* IgY (prepared in the laboratory, purity 85%, and valence combined recombinant human IL-1*β*: 1 : 3200/mg protein) [4] and an OVA solution for seven days by instilling the nostrils with 0.2 mL of an OVA solution after instilling the nostrils with 0.2 mL of 0.1% anti-IL-1*β* IgY (0.1 mL/each nostril). (5) The 0.1% combined anti-IL-1*β*/TNF-*α* IgY treatment group (group Z_4_) (*n* = 18) was treated with 0.1% of combined anti-IL-1*β* and TNF-*α* IgY antibodies (half of the 0.1% anti-IL-1*β* IgY and half of the anti-TNF-*α* IgY were mixed together to produce the 0.1% combined anti-IL-1*β* IgY and anti-TNF-*α* IgY solution) [1] and an OVA solution for seven days by instilling the nostrils with 0.2 mL of an OVA solution after instilling the nostrils with 0.2 mL of 0.1% combined anti-IL-1*β* and TNF-*α* IgY (0.1 mL/each nostril). The above IgY preparations do not contain LPS and ovalbumin. (6) The fluticasone propionate treatment group (the positive control, group Z_5_) (*n* = 17) was treated with a fluticasone propionate suspension (0.05%, GlaxoSmithKline, PLC, UK) and an OVA solution for seven days by instilling the nostrils with 0.2 mL of an OVA solution after instilling the nostrils with 0.2 mL of the fluticasone propionate suspension (0.1 mL/each nostril). The blood, NLF, and BALF were obtained from the guinea pigs of all experimental groups at 2, 4, and 8 hours after last treatment and challenge. The inflammatory cells were examined in the PB, NLF, BALF, nasal mucosa, and lung tissues.

### 2.3. Blood, NLF, and BALF Collection

The guinea pigs were anesthetized using 10% chloral hydrate via intraperitoneal injection. The plasma, NLF, and BALF collection described by Guo-Zhu et al. [1] and Wei-xu et al. [4] was used. The precipitated cells were smeared. The blood, NLF, and BALF were collected at 2 (*n* = three to six guinea pigs in each group), 4 (*n* = four to six guinea pigs in each group), and 8 (*n* = three to six guinea pigs in each group) hours in random order. In brief, the guinea pig' head was fixed to lie on one's back, a cannula attached to the nasal cavity of one nostrils was connected to a 5.0 mL syringe with 2.0 mL 0.9% saline, 0.9% saline slowly was perfused into the nasal cavity at a rate of 0.1 mL/min, and nasal lavage liquid was collected with micropipette on the other nostrils. NLF was centrifuged and supernatant was stored at −80°C. Each heart was exposed and heart blood was withdrawn using a 5.0 mL syringe with EDTA. Blood was centrifuged and plasma was stored at −80°C. The left trachea was exposed and cannulated with silicone tubing attached to a 23-gauge needle on a 5.0 mL syringe. After instillation of 2.0 mL of sterile PBS through the trachea into the lung, BALF was withdrawn and centrifuged. Supernatants were stored at −80°C.

### 2.4. Cell Staining

The cell staining methods described by Wei-xu et al. [4] were used. In brief, drops of the blood and precipitated cells in the BALF were placed onto a slide and smeared. After drying at room temperature, methylene blue was dripped onto the slide. The cells were fixed and stained for 1-2 min, and eosin was dripped onto the slide and the cells were stained for an additional 10 min. The slide was then rinsed with distilled water. More than 200 cells were randomly counted in a high power field, and eosinophils, neutrophils, lymphocytes, macrophages, monocytes, and basophils were counted.

### 2.5. Pathological Examination of the Nasal Mucosa and Lung Tissues

The guinea pigs were anesthetized using 10% chloral hydrate via intraperitoneal injection and were sacrificed. The nasal mucosa and right lung were removed. Hematoxylin-eosin (HE) staining was performed according to the standard procedure [4]. The pathological inflammatory reactions were observed in HE-stained tissue sections using a high magnification lens and a microscope. The eosinophils were randomly counted in high magnification five fields of HE-stained sections using a microscope, and the percentage of eosinophils in the inflammatory cells was determined.

### 2.6. Statistical Analysis

The experimental data were expressed as the means ± standard deviation (*X* ± *S*). The tests for normal distribution and the homogeneity of the variances were performed for the different groups at each time point. If the experimental data conformed to a normal distribution and homogeneity of variance, the comparison between the groups was performed using one-way ANOVA, and post hoc tests were used for comparisons between two groups. If the experimental data did not fit a normal distribution or homogeneity of variance, a nonparametric test was used to compare the differences between groups. *P* < 0.05 was considered statistically significant.

## 3. Results 

### 3.1. Pathology of Nasal Mucosa Inflammation

**Figure 1:**
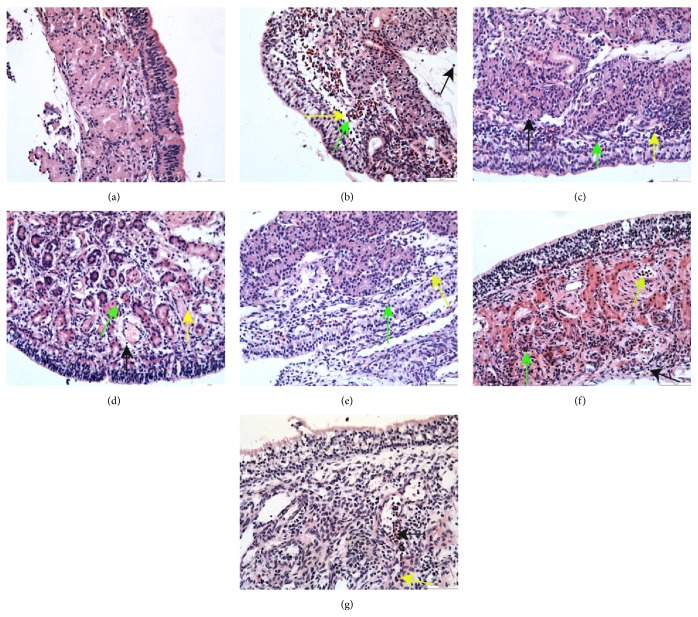
HE staining of nasal mucosal sections at 8 h in guinea pigs of the healthy control and different treated AR groups (200x). (a) The healthy control group (group C); (b) the AR model group (group M); (c) the 0.1% nonspecific IgY-treated group (group Z_1_); (d) the 0.1% anti-TNF-*α*-treated group (group Z_2_); (e) the 0.1% anti-IL-1*β*-treated group (group Z_3_); (f) the 0.1% anti-IL-1*β*/TNF-*α*-treated group (group Z_4_); and (g) the fluticasone propionate-treated group (group Z_5_).* Eosinophils* infiltration (the green arrows);* neutrophils* infiltration (the yellow arrows);* lymphocytes* infiltration (the black arrows).

Lymphocyte infiltration was occasionally observed in the lamina propria of the nasal mucosa at 2, 4, and 8 hours, and the mucosal epithelial cell lamina was intact in the healthy guinea pigs (Figure 1(a); E-Figures 1-2 A (in Supplementary Material available online at http://dx.doi.org/10.1155/2016/3128182)). However, in the AR model guinea pigs and 0.1% nonspecific IgY-treated guinea pigs, a large number of eosinophils, neutrophils, and lymphocytes infiltrated into the mucosal epithelial cell lamina and the lamina propria of the nasal mucosa. There was edema in the lamina propria of the nasal mucosa, and the epithelial cells had fallen off in the mucosal epithelial cell lamina at 2, 4, and 8 hours (Figures 1(b)-1(c); E-Figures 1-2 B-C). In the 0.1% anti-TNF-*α*-treated and 0.1% anti-IL-1*β*-treated guinea pigs, many eosinophils, neutrophils, and lymphocytes infiltrated into the mucosal epithelial cell lamina and the lamina propria of the nasal mucosa. There was edema in the lamina propria of the nasal mucosa, and some of the epithelial cells had fallen off of the mucosal epithelial cell lamina at 2 and 4 hours. By 8 h, we observed reduced eosinophil, neutrophil, and lymphocyte infiltration, an alleviated pathological inflammatory response, and that the mucosal epithelial cell lamina was more intact in the nasal mucosa (Figures 1(d)-1(e); E-Figures 1-2 D-E). In the 0.1% anti-TNF-*α*/IL-1*β*-treated and fluticasone propionate-treated guinea pigs, a small number of eosinophils, neutrophils, and lymphocytes infiltrated into the mucosal epithelial cell lamina and the lamina propria of the nasal mucosa, the edema in the lamina propria of the nasal mucosa was reduced, and the mucosal epithelial cell lamina was more intact in the nasal mucosa at 2 h. Eosinophil, neutrophil, and lymphocyte infiltration was further reduced, and edema was further alleviated at 4 h; furthermore, eosinophil, neutrophil, and lymphocyte infiltration was significantly reduced and edema was absent at 8 h. Moreover, compared to the 0.1% anti-TNF-*α*-treated and 0.1% anti-IL-1*β*-treated guinea pigs, the 0.1% anti-TNF-*α*/IL-1*β*-treated guinea pigs showed less severe tissue damage, particularly at 8 h (Figures 1(f)-1(g); E-Figures 1-2 F-G).

### 3.2. Pathology of Lung Tissue Inflammation

**Figure 2:**
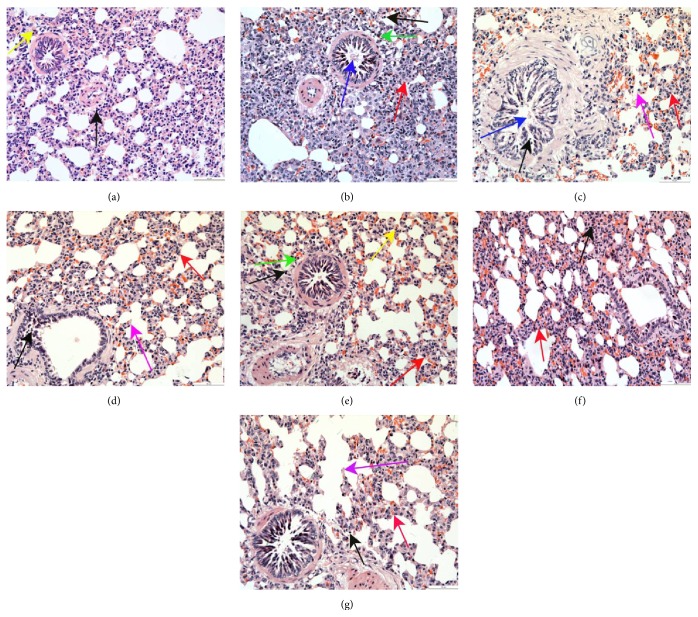
HE staining of lung tissue sections at 8 h in guinea pigs of the healthy control and different treated AR groups (200x). (a) The healthy control group (group C); (b) the AR model group (group M); (c) the 0.1% nonspecific IgY-treated group (group Z_1_); (d) the 0.1% anti-TNF-*α*-treated group (group Z_2_); (e) the 0.1% anti-IL-1*β*-treated group (group Z_3_); (f) the 0.1% anti-IL-1*β*/TNF-*α*-treated group (group Z_4_); and (g) the fluticasone propionate-treated group (group Z_5_).* Eosinophils* infiltration (the green arrows);* neutrophils* infiltration (the yellow arrows);* lymphocytes* infiltration (the black arrows);* broadening* of the alveolar septa (the red arrows);* fracture* of alveolar septa (the violet arrows).* The partial drop* of the number of bronchial mucosal epithelial cells and the discharge of intrabronchial secreta (the blue arrows).

Inflammatory cell, primarily lymphocytes, infiltration was occasionally observed in the lung tissues at 2, 4, and 8 hours, and the lung tissue structure and morphology were normal in the healthy guinea pigs (Figure 2(a); E-Figures 3-4 A). However, in the AR model guinea pigs and the 0.1% nonspecific IgY-treated guinea pigs, a large number of eosinophils, neutrophils, and lymphocytes were observed in the alveolar septa and around the bronchioli and blood vessels. We observed pulmonary interstitial edema, damage to the alveolar tube, marked thickening and fracture of alveolar septa, a partial decreased in the bronchial mucosal epithelial cells, and thickening of the bronchial smooth muscle at 2 and 4 hours. By 8 h, more significant pathological inflammatory responses, pulmonary consolidation, and discharge of the intrabronchial secreta were observed in the AR model guinea pigs. Bronchial mucosa and pulmonary interstitial edema, fracture of alveolar septa and alveolar fusion, and bronchial smooth muscle hyperplasia were more visible in the 0.1% nonspecific IgY-treated guinea pigs (Figures 2(b)-2(c); E-Figures 3-4 B-C). In the 0.1% anti-TNF-*α*-treated and 0.1% anti-IL-1*β*-treated guinea pigs, many eosinophils, neutrophils, and lymphocytes were observed in the alveolar septa and around the bronchiole vessel and the damage to the alveolar tube was reduced; however, the bronchial mucosa, pulmonary interstitial edema, and thickening of the alveolar septa and bronchial smooth muscle were reduced compared to the AR model guinea pigs and the 0.1% nonspecific IgY-treated guinea pigs at 2 and 4 hours. By 8 h, the pathological inflammatory responses were more reduced; the bronchial mucosa and pulmonary interstitial edema, endobronchial exudate and eosinophils, neutrophils, and lymphocytes in the alveolar septa and around the bronchioli vessel were significantly alleviated and reduced compared to 2 and 4 h (Figures 2(d)-2(e); E-Figures 3-4 D-E). In the 0.1% anti-TNF-*α*/IL-1*β*-treated and fluticasone propionate-treated guinea pigs, a small number of eosinophils, neutrophils, and lymphocytes were observed in the alveolar septa and around the bronchioli vessels, and we observed a mild broadening of the alveolar septa and a few eosinophils, neutrophils, and lymphocytes in the bronchial cavity at 2 and 4 hours. By 8 h, we observed a milder broadening of the alveolar septa; fewer inflammatory cells, primarily lymphocytes, in the alveolar septa and around the bronchioli vessel; a clear bronchial cavity; and further reduced pathological inflammatory responses compared to 2 h and 4 h. Compared to the 0.1% anti-TNF-*α*-treated and 0.1% anti-IL-1*β*-treated guinea pigs, the 0.1% anti-TNF-*α*/IL-1*β*-treated and fluticasone propionate-treated guinea pigs showed less severe tissue damage (Figures 2(f)-2(g); E-Figures 3-4 F-G).

### 3.3. Variation in the Number of Eosinophils in the Nasal Mucosa and Lung Tissues

**Figure 3:**
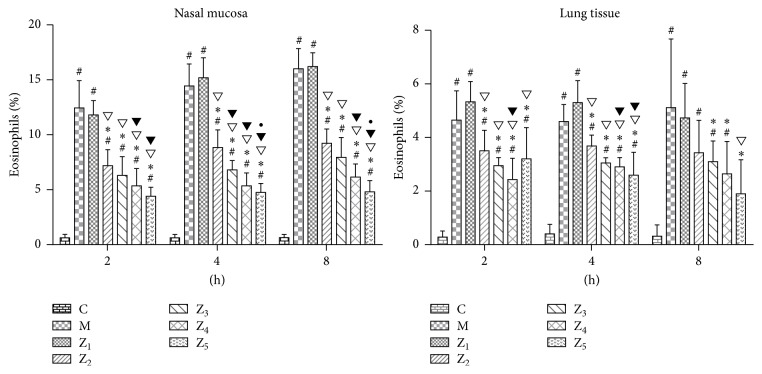
Percentage of eosinophils (*X* ± *S*  %) in the nasal mucosa and lung tissues in guinea pigs of the healthy control and different treated AR groups. C, the healthy control group (group C); M, the AR model group (group M); Z_1_, the 0.1% nonspecific IgY-treated group (group Z_1_); Z_2_, the 0.1% anti-TNF-*α*-treated group (group Z_2_); Z_3_, the 0.1% anti-IL-1*β*-treated group (group Z_3_); Z_4_, the 0.1% anti-IL-1*β*/TNF-*α*-treated group (group Z_4_); and Z_5_, the fluticasone propionate-treated group (group Z_5_); *n* = 3~6 per group; #: *P* < 0.05 compared with the healthy control group (group C); *∗*: *P* < 0.05 compared with the AR model group (group M); ▽: *P* < 0.05 compared with the 0.1% nonspecific IgY-treated group (group Z_1_); ▼: *P* < 0.05 compared with the 0.1% anti-TNF-*α*-treated group (group Z_2_); •: *P* < 0.05 compared with the 0.1% anti-IL-1*β*-treated group (group Z_3_).

We found that the number of eosinophils in the nasal mucosa was significantly increased in the AR model, 0.1% nonspecific IgY-treated, 0.1% anti-TNF-*α*-treated, 0.1% anti-IL-1*β*-treated, 0.1% anti-TNF-*α*/IL-1*β*-treated, and fluticasone propionate-treated guinea pigs compared to the healthy guinea pigs at 2, 4, and 8 hours (*P* < 0.05), while the number of eosinophils in the lung tissues was also significantly increased in these same groups compared to the healthy guinea pigs at 2 and 4 hours (*P* < 0.05). In the 0.1% anti-TNF-*α*-treated, 0.1% anti-IL-1*β*-treated, 0.1% anti-TNF-*α*/IL-1*β*-treated, and fluticasone propionate-treated guinea pigs, the number of eosinophils in the nasal mucosa was significantly reduced at 2, 4, and 8 hours compared to the AR model guinea pigs and the 0.1% nonspecific IgY-treated guinea pigs and was reduced in the lung tissues at 2 and 4 hours (*P* < 0.05). In the 0.1% anti-IL-1*β*-treated, 0.1% anti-TNF-*α*/IL-1*β*-treated, and fluticasone propionate-treated guinea pigs, the number of eosinophils in the lung tissues was significantly reduced at 8 h compared to the AR model guinea pigs; in the fluticasone propionate-treated guinea pigs, the number of eosinophils in the lung tissues was significantly reduced at 8 h compared to the 0.1% nonspecific IgY-treated guinea pigs. The number of eosinophils was significantly reduced in the 0.1% anti-TNF-*α*/IL-1*β*-treated and fluticasone propionate-treated guinea pigs at 2, 4, and 8 hours and in the 0.1% anti-IL-1*β*-treated guinea pigs at 4 h compared to the 0.1% anti-TNF-*α*-treated guinea pigs in nasal mucosa. The number of eosinophils was significantly reduced in the lung tissues of the 0.1% anti-TNF-*α*/IL-1*β*-treated guinea pigs at 2 and 4 hours and the fluticasone propionate-treated guinea pigs at 4 hours compared to the 0.1% anti-TNF-*α*-treated guinea pigs (*P* < 0.05) (Figure 3). However, we did not observe a significant difference between the 0.1% anti-TNF-*α*/IL-1*β*-treated guinea pigs and the fluticasone propionate-treated guinea pigs (Figure 3).

### 3.4. Variations in the Number of Eosinophils in the PB, NLF, and BALF

**Figure 4:**
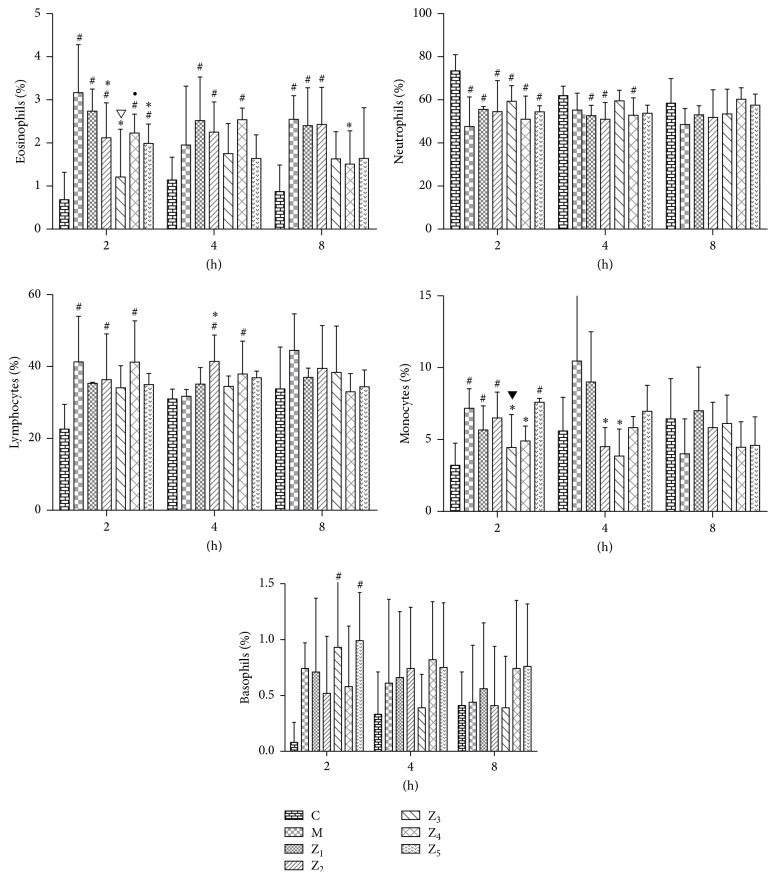
Percentage of different inflammatory cells (*X* ± *S*  %) in the PB in guinea pigs of the healthy control and the different treated AR groups. C, the healthy control group (group C); M, the AR model group (group M); Z_1_, the 0.1% nonspecific IgY-treated group (group Z_1_); Z_2_, the 0.1% anti-TNF-*α*-treated group (group Z_2_); Z_3_, the 0.1% anti-IL-1*β*-treated group (group Z_3_); Z_4_, the 0.1% anti-IL-1*β*/TNF-*α*-treated group (group Z_4_); and Z_5_, the fluticasone propionate-treated group (group Z_5_); *n* = 3~6 per group; #: *P* < 0.05 compared with the healthy control group (group C); *∗*: *P* < 0.05 compared with the AR model group (group M); ▽: *P* < 0.05 compared with the 0.1% nonspecific IgY-treated group (group Z_1_); ▼: *P* < 0.05 compared with the 0.1% anti-TNF-*α*-treated group (group Z_2_); •: *P* < 0.05 compared with the 0.1% anti-IL-1*β*-treated group (group Z_3_).

In the PB, the number of eosinophils was significantly reduced at 2 h in the 0.1% anti-IL-1*β*-treated and fluticasone propionate-treated guinea pigs and at 8 h in the 0.1% anti-IL-1*β*/TNF-*α*-treated guinea pigs compared to the AR model guinea pigs (*P* < 0.05). The number of eosinophils was significantly reduced at 2 h in the 0.1% anti-IL-1*β*-treated guinea pigs compared to the 0.1% nonspecific IgY-treated guinea pigs (*P* < 0.05). However, we did not observe a significant difference between the 0.1% anti-IL-1*β*/TNF-*α*-treated guinea pigs and the fluticasone propionate-treated guinea pigs (Figure 4). These results indicate that the curative effect of a topical intranasal 0.1% anti-IL-1*β*/TNF-*α* IgY instilling in AR guinea pigs is limited for systemic symptoms.

**Figure 5:**
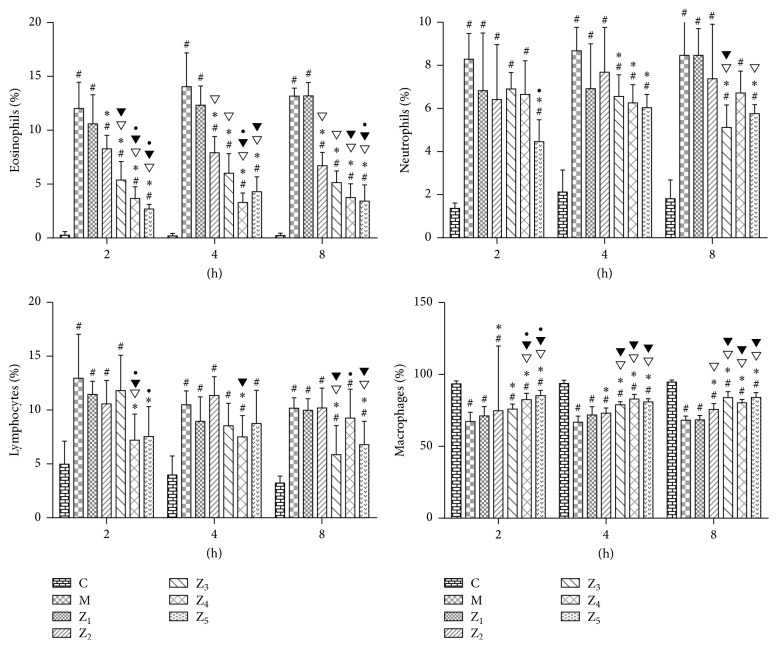
Percentage of different inflammatory cells (*X* ± *S*  %) in the NLF in guinea pigs of the healthy control and the different treated AR groups. C, the healthy control group (group C); M, the AR model group (group M); Z_1_, the 0.1% nonspecific IgY-treated group (group Z_1_); Z_2_, the 0.1% anti-TNF-*α*-treated group (group Z_2_); Z_3_, the 0.1% anti-IL-1*β*-treated group (group Z_3_); Z_4_, the 0.1% anti-IL-1*β*/TNF-*α*-treated group (group Z_4_); and Z_5_, the fluticasone propionate-treated group (group Z_5_); *n* = 3~6 per group; #: *P* < 0.05 compared with the healthy control group (group C); *∗*: *P* < 0.05 compared with the AR model group (group M); ▽: *P* < 0.05 compared with the 0.1% nonspecific IgY-treated group (group Z_1_); ▼: *P* < 0.05 compared with the 0.1% anti-TNF-*α*-treated group (group Z_2_); •: *P* < 0.05 compared with the 0.1% anti-IL-1*β*-treated group (group Z_3_).

In the NLF, the number of eosinophils was significantly reduced in the 0.1% anti-TNF-*α*-treated, 0.1% anti-IL-1*β*-treated, 0.1% anti-IL-1*β*/TNF-*α*-treated, and fluticasone propionate-treated guinea pigs at 2, 4, and 8 hours compared to the AR model guinea pigs (*P* < 0.05). The number of eosinophils was also significantly reduced in the 0.1% anti-IL-1*β*-treated, 0.1% anti-IL-1*β*/TNF-*α*-treated, and fluticasone propionate-treated guinea pigs at 2, 4, and 8 hours and the 0.1% anti-TNF-*α*-treated guinea pigs at 4 and 8 hours compared to the 0.1% nonspecific IgY-treated guinea pigs (*P* < 0.05) (Figure 5). It was interesting that the number of eosinophils was significantly reduced in the 0.1% anti-IL-1*β*/TNF-*α*-treated and fluticasone propionate-treated guinea pigs at 2, 4, and 8 hours and in the 0.1% anti-IL-1*β*-treated guinea pigs at 2 h compared to the 0.1% anti-TNF-*α*-treated guinea pigs. The number of eosinophils was also significantly reduced in the 0.1% anti-IL-1*β*/TNF-*α*-treated guinea pigs at 2 and 4 hours and in the fluticasone propionate-treated guinea pigs at 2 and 8 hours compared to the 0.1% anti-IL-1*β*-treated guinea pigs (*P* < 0.05) (Figure 5). However, we did not observe a significant difference between the 0.1% anti-IL-1*β*/TNF-*α*-treated guinea pigs and the fluticasone propionate-treated guinea pigs. These results indicate that the curative effect of a topical intranasal 0.1% anti-IL-1*β*/TNF-*α* IgY treatment in AR guinea pigs is remarkable and definitely prevented pathological inflammatory responses in the nasal mucosa of the OVA-induced AR guinea pigs.

**Figure 6:**
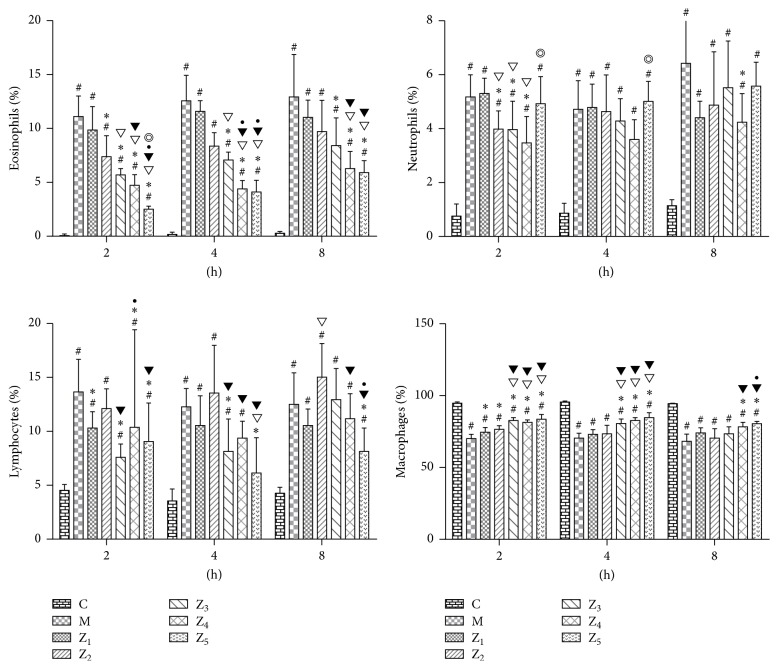
Percentage of different inflammatory cells (*X* ± *S*  %) in the BALF in guinea pigs of the healthy control and the different treated AR groups. C, the healthy control group (group C); M, the AR model group (group M); Z_1_, the 0.1% nonspecific IgY-treated group (group Z_1_); Z_2_, the 0.1% anti-TNF-*α*-treated group (group Z_2_); Z_3_, the 0.1% anti-IL-1*β*-treated group (group Z_3_); Z_4_, the 0.1% anti-IL-1*β*/TNF-*α*-treated group (group Z_4_); and Z_5_, the fluticasone propionate-treated group (group Z_5_); *n* = 3~6 per group; #: *P* < 0.05 compared with the healthy control group (group C); *∗*: *P* < 0.05 compared with the AR model group (group M); ▽: *P* < 0.05 compared with the 0.1% nonspecific IgY-treated group (group Z_1_); ▼: *P* < 0.05 compared with the 0.1% anti-TNF-*α*-treated group (group Z_2_); •: *P* < 0.05 compared with the 0.1% anti-IL-1*β*-treated group (group Z_3_); *◎*: *P* < 0.05 compared with the 0.1% anti-IL-1*β*/TNF-*α*-treated group (group Z_4_).

In the BALF, the number of eosinophils was significantly reduced at 2 h in the 0.1% anti-IL-1*β*-treated, 0.1% anti-IL-1*β*/TNF-*α*-treated, and fluticasone propionate-treated guinea pigs compared to the AR model and 0.1% nonspecific IgY-treated guinea pigs and at 2 h in the 0.1% anti-IL-1*β*/TNF-*α*-treated and fluticasone propionate-treated guinea pigs compared to the 0.1% anti-TNF-*α*-treated guinea pigs (*P* < 0.05). The number of eosinophils was significantly reduced at 4 h in the 0.1% anti-IL-1*β*-treated, 0.1% anti-IL-1*β*/TNF-*α*-treated, and fluticasone propionate-treated guinea pigs compared to the AR model and 0.1% nonspecific IgY-treated guinea pigs and at 4 h in the 0.1% anti-IL-1*β*/TNF-*α*-treated and fluticasone propionate-treated guinea pigs compared to the 0.1% anti-TNF-*α*-treated and 0.1% anti-IL-1*β*-treated guinea pigs (*P* < 0.05). The number of eosinophils was significantly reduced at 8 h in the 0.1% anti-IL-1*β*-treated, 0.1% anti-IL-1*β*/TNF-*α*-treated, and fluticasone propionate-treated guinea pigs compared to the AR model guinea pigs and at 8 h in the 0.1% anti-IL-1*β*/TNF-*α*-treated and fluticasone propionate-treated guinea pigs compared to the 0.1% nonspecific IgY-treated and 0.1% anti-TNF-*α*-treated guinea pigs (*P* < 0.05) (Figure 6). However, we did not observe a significant difference between the 0.1% anti-IL-1*β*/TNF-*α*-treated guinea pigs and the fluticasone propionate-treated guinea pigs. These results indicate that AR and allergic asthma represent a continuum of respiratory allergic diseases. The topical intranasal anti-IL-1*β*/TNF-*α* IgY treatment can effectively prevent pathological inflammatory responses in lung tissues of OVA-induced AR guinea pigs induced by OVA.

## 4. Discussion

The local recruitment and activation of leukocytes and the productions of inflammatory cytokines and T helper 2 (Th2) cytokines in allergic rhinitis contribute to the development of late-phase pathologic allergic reactions. Our results and other scholars all have demonstrated increases in inflammatory cells infiltration, particularly eosinophils, in the nasal mucosa in the OVA-induced AR model guinea pigs [2] and mice [3] and in eosinophils infiltration in the nasal mucosa of allergic rhinitis patients [9, 10]. Now, our data showed that the 0.1% anti-IL-1*β*/TNF-*α* IgY intranasal instillation therapy effectively alleviated the allergic late-phase reaction in the nasal mucosa of OVA-challenged guinea pigs. In 0.1% anti-IL-1*β*/TNF-*α* IgY-treated AR guinea pigs, eosinophil, neutrophil, and lymphocyte infiltration was significantly reduced and edema was absent compared to the 0.1% anti-TNF-*α* IgY-treated and 0.1% anti-IL-1*β* IgY-treated AR guinea pigs; the 0.1% anti-IL-1*β*/TNF-*α* IgY-treated animals showed less inflammatory cell infiltration and less severe pathological tissue damage in the nasal mucosa, particularly at 8 h (*P* < 0.05). These interesting results not only indicate that the curative effect of a topical intranasal 0.1% anti-IL-1*β*/TNF-*α* IgY treatment remarkably and definitely prevented the pathological inflammatory responses in OVA-induced AR guinea pigs but also demonstrate that the proinflammatory cytokines IL-1*β* and TNF-*α* are initiating or accelerating factors in allergic inflammation. These proinflammatory cytokines must also be blocked simultaneously. Other scholars also have demonstrated that the TNF-*α* inhibitor infliximab reduced the allergic symptoms and eosinophilic infiltration into the nasal mucosa and suppressed the local Th2 cytokine transcription in the nasal mucosa of the OVA-induced AR mice [11].

Our results showed that the numbers of eosinophils, neutrophils, and lymphocytes in the lung and BALF were increased and pulmonary pathological changes were also observed in the AR model guinea pigs. Other scholars also demonstrated that the eosinophilic infiltrates in the lung and granulocytes in the BALF were concomitantly increased in the OVA-exposed BALB/c AR mouse model [12]. The total numbers of inflammatory cells, primarily eosinophils, in the BALF and pulmonary tissue and epithelial damage were increased in OVA-sensitized AR rats [5]. Airway inflammation in rhinitic subjects was characterized by an increase in submucosal eosinophils at an intermediate level between healthy controls and asthmatics [13]. Oka et al. found that 66.9% patients with asthma suffered from allergic rhinitis. This observational study of patients with atopy indicated that the ongoing allergic rhinitis is related to worsening of asthma by enhancing the lower airway inflammation [14]. Brown et al. demonstrated that airway inflammation in rhinitic subjects was characterized by an increase in submucosal eosinophils, mast cells, and the mRNA expression of TNF-*α* at an intermediate level between healthy and asthmatics. These results indicated that allergic rhinitis and asthma represent a continuum of atopic disease [13]. Now, our data showed that except the nasal mucosa inflammation, there were a large number of inflammatory cells in the alveolar septa and around the bronchioli and blood vessels. The pulmonary interstitial edema, damage to the alveolar tube, marked thickening and fracture of alveolar septa, decreasing of the bronchial mucosal epithelial cells, thickening of the bronchial smooth muscle, and pulmonary consolidation were observed in the lung tissues in the AR model guinea pigs. However, the intranasal instillation of 0.1% anti-IL-1*β*/TNF-*α* IgY effectively alleviated the allergic late-phase reaction in the lung tissues from OVA-challenged guinea pigs. Compared to the 0.1% anti-TNF-*α* IgY-treated AR model guinea pigs, the 0.1% anti-IL-1*β*/TNF-*α* IgY-treated animals showed less inflammatory cell infiltration (*P* < 0.05) and less severe pathological tissue damage, and the numbers of eosinophils, neutrophils, and lymphocytes were significantly reduced in the 0.1% anti-IL-1*β*/TNF-*α*-treated guinea pigs compared to the AR model guinea pigs in the BALF (*P* < 0.05). These interesting results also indicate that the proinflammatory cytokines IL-1*β* and TNF-*α* are initiating or accelerating factors in allergic inflammation and that both proinflammatory cytokines must simultaneously be blocked. The topical intranasal 0.1% anti-IL-1*β*/TNF-*α* IgY treatment can effectively prevent pathological inflammatory responses in the lung tissues of OVA-induced AR guinea pigs. At present, the glucocorticoid is still the most effective therapeutic agents in the treatment of allergic rhinitis and asthma. However, it is interesting that therapeutic effects between the 0.1% anti-TNF-*α*/IL-1*β*-treated and the fluticasone propionate-treated AR guinea pigs had not a significant difference. The anti-IL-1*β*/TNF-*α* IgY blocks the effective stage of the immune response and fluticasone propionate (glucocorticoid) inhibits the initial stage of the immune response. Glucocorticoid use for a long time has serious side effects, but IgY antibody has no definite side effects. Orally administered IgY against pseudomonas aeruginosa prevents pulmonary pseudomonas aeruginosa infections in patients with cystic fibrosis, and no negative side effects of IgY treatment have been noted during these 10 years [15].

Inflammatory cytokines can interact to aggravate allergic inflammatory pathological responses, and the cytokine network of inflammatory cytokine interactions is very complicated during the development of allergic inflammatory pathology [16]. Mice lacking IL-1R failed to mount a Th2 immune response and did not develop asthma to HDM, IL-1*α* acted in an autocrine manner to trigger the release of DC-attracting chemokines, IL-33, and allergic sensitization to HDM was abolished in vivo when IL-1*α* was neutralized. These findings place IL-1*α* upstream in the cytokine cascade, leading to epithelial and DC activation in response to an inhaled HDM allergen [17]. In AR mice, eosinophil infiltration in the nasal mucosa was significantly restricted in TNF-alpha(−/−) mice compared with in TNF-alpha(+/+) mice after OVA sensitization [18]. The nasal lavage fluid levels of MPO post-TNF-*α* challenge were increased in patients with allergic rhinitis. TNF-*α* increased the number of subepithelial neutrophils [19]. TNF-*α* markedly enhanced the effect of TGF-*β*1 on the epithelial-mesenchymal transition [20]. The above research results from the cytokine interaction networks of allergic animals and patients are consistent with our previous [1] and current results. These interesting results indicate that proinflammatory cytokines IL-1*β* and TNF-*α* are initiating factors or acceleration factors in allergic inflammation, that both proinflammatory cytokines are equally important in the cytokine interaction network, and that both proinflammatory cytokines must simultaneously be blocked.

In summary, the intranasal treatment using anti-IL-1*β*/anti-TNF-*α* IgY can significantly relieve allergic inflammation in the nasal mucosa and lung tissues and decrease eosinophils in the peripheral blood, nasal lavage and bronchoalveolar lavage fluid, nasal mucosa, and lung tissues in guinea pigs with allergic rhinitis induced by OVA. The combined blockade of IL-1*β* and TNF-*α* by the intranasal instillation of 0.1% anti-IL-1*β*/TNF-*α* IgY could be a potential alternative strategy for preventing and treating allergic rhinitis.

## Supplementary Material

Pathology of nasal mucosa inflammation: Lymphocyte infiltration was occasionally observed in the lamina propria of the nasal mucosa and the mucosal epithelial cell lamina were intact at 2 and 4 hours in the healthy guinea pigs (E-Fig. 1-2 A). However, in the AR model guinea pigs and 0.1% non-specific IgY-treated guinea pigs, a large number of eosinophils, neutrophils and lymphocytes infiltrated into the mucosal epithelial cell lamina and the lamina propria of the nasal mucosa at 2 and 4 hours. There was edema in the lamina propria of the nasal mucosa, and the epithelial cells had fallen off in the mucosal epithelial cell lamina at 2 and 4 hours in the AR model guinea pigs and 0.1% non-specific IgY-treated guinea pigs (E-Fig. 1-2 B-C). In the 0.1% anti-TNF-*α*-treated and 0.1% anti-IL-1*β*-treated guinea pigs, many eosinophils, neutrophils and lymphocytes infiltrated into the mucosal epithelial cell lamina and the lamina propria of the nasal mucosa at 2 and 4 hours. There was edema in the lamina propria of the nasal mucosa in the 0.1% anti-TNF-*α*-treated and 0.1% anti-IL-1*β*-treated guinea pigs at 2 and 4 hours. Some of the epithelial cells of the mucosal epithelial cell lamina had fallen off at 2 and 4 hours in the 0.1% anti-TNF-*α*-treated and 0.1% anti-IL-1*β*-treated guinea pigs at 2 and 4 hours. The inflammatory response was heavier in the lamina propria of the nasal mucosa at 4 than at 2 hours in the 0.1% anti-TNF-*α*-treated and 0.1% anti-IL-1*β*-treated guinea pigs (E-Fig. 1-2 D-E). In the 0.1% anti-TNF-*α*/IL-1*β*-treated and fluticasone propionate-treated guinea pigs, a small number of eosinophils, neutrophils and lymphocytes infiltrated into the mucosal epithelial cell lamina and the lamina propria of the nasal mucosa, and the mucosal epithelial cell lamina was more intact in the nasal mucosa at 2 h. The edema in the lamina propria of the nasal mucosa was heavier in the lamina propria of the nasal mucosa at 2 h in the 0.1% anti-TNF-*α*/IL-1*β*-treated guinea pigs than in the fluticasone propionate-treated guinea pigs. Eosinophil, neutrophil and lymphocyte infiltration was further reduced, edema was further alleviated, and the mucosal epithelial cell lamina was more intact at 4 h in the 0.1% anti-TNF-*α*/IL-1*β*-treated and fluticasone propionate-treated guinea pigs. However, the lamina propria of the nasal mucosa was loose at 4 h in the fluticasone propionate-treated guinea pigs (E-Fig. 1-2 F-G).
Pathology of lung tissue inflammation: Lymphocytes infiltration was occasionally observed in the lung tissues and the lung tissue structure and morphology were normal at 2 and 4 hours in the healthy guinea pigs (E-Fig. 3-4 A). However, in the AR model guinea pigs and the 0.1% non-specific IgY-treated guinea pigs, a large number of eosinophils, neutrophils and lymphocytes were observed in the alveolar septa and around the bronchioli and blood vessels at 2 and 4 hours. We observed pulmonary interstitial edema, damage to the alveolar tube, alveoli fusion, marked thickening and fracture of alveolar septa, a partial decreased in the bronchial mucosal epithelial cells, and thickening of the bronchial smooth muscle at 2 and 4 hours in the AR model guinea pigs and the 0.1% non-specific IgY-treated guinea pigs (E-Fig. 3-4 B-C). In the 0.1% anti-TNF-*α*-treated and 0.1% anti-IL-1*β*-treated guinea pigs, many eosinophils, neutrophils and lymphocytes were observed in the alveolar septa and around the bronchioli vessel at 2 and 4 hours. The damage to the alveolar tube was reduced at 2 and 4 hours in the 0.1% anti-TNF-*α*-treated and 0.1% anti-IL-1*β*-treated guinea pigs. the bronchial mucosa, pulmonary interstitial edema, and thickening of the alveolar septa and bronchial smooth muscle were reduced compared to the AR model guinea pigs and the 0.1% non-specific IgY-treated guinea pigs at 2 and 4 hours (E-Fig. 3-4 D-E). In the 0.1% anti-TNF-*α*/IL-1*β*-treated and fluticasone propionate-treated guinea pigs, a small number of eosinophils, neutrophils and lymphocytes were observed in the alveolar septa and around the bronchioli vessels, and we observed a mild broadening of the alveolar septa and a few eosinophils, neutrophils and lymphocytes in the bronchial cavity at 2 and 4 hours. Compared to the 0.1% anti-TNF-*α*-treated and 0.1% anti-IL-1*β*-treated guinea pigs, the 0.1% anti-TNF-*α*/IL-1*β*-treated and fluticasone propionate-treated guinea pigs showed less severe tissue damage (E-Fig. 3-4 F-G).

## Abbreviations

AR: Allergic rhinitis
BALF: Bronchoalveolar lavage fluid
IgE: Immunoglobulin E
IgY: Immunoglobulin yolk
IL-1*β*: Interleukin-1 beta
IL-5: Interleukin-5
IL-9: Interleukin-9
IL-13: Interleukin-13
IL-18: Interleukin-18
IL-22: Interleukin-22
IL-33: Interleukin-33
Infliximab: Anti-TNF-*α* antibody
NLF: Nasal lavage fluid
OVA: Ovalbumin
PB: Peripheral blood
TGF-*β*_1_: Transforming growth factor-beta 1
Th2: T helper type 2
TNF-*α*: Tumor necrosis factor-alpha.
