# Supplementary figures and images for: Anti-Interleukin-1 Beta/Tumor Necrosis Factor-Alpha IgY Antibodies Reduce Pathological Allergic Responses in Guinea Pigs with Allergic Rhinitis
Source: Mediators Inflamm. 2016 Mar 7;2016:3128182. doi: 10.1155/2016/3128182 (PMC4800104; doi:10.1155/2016/3128182)

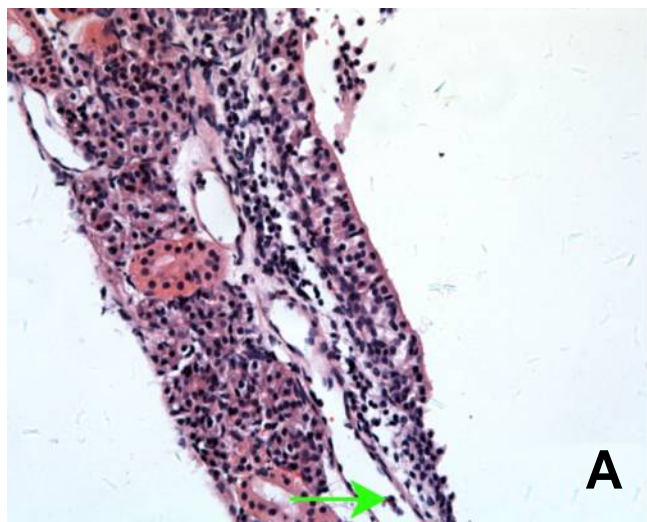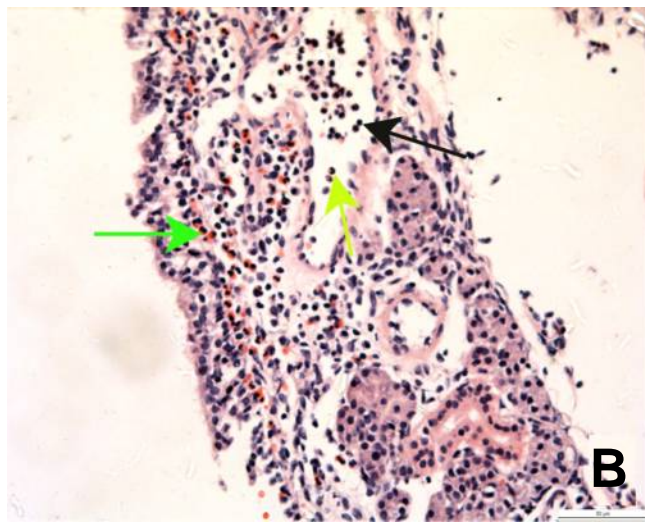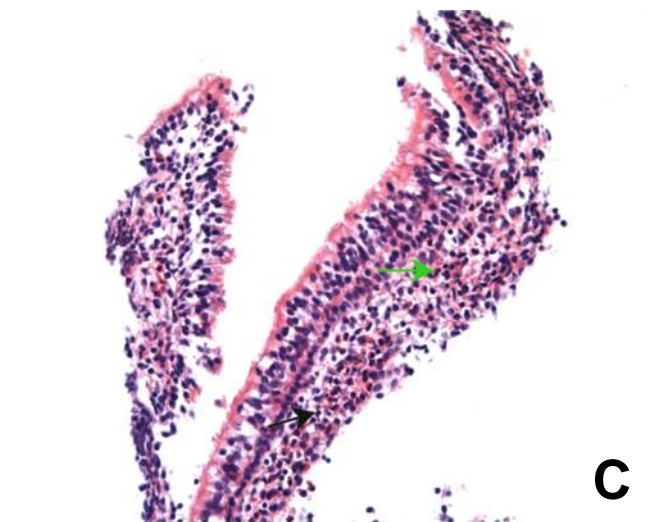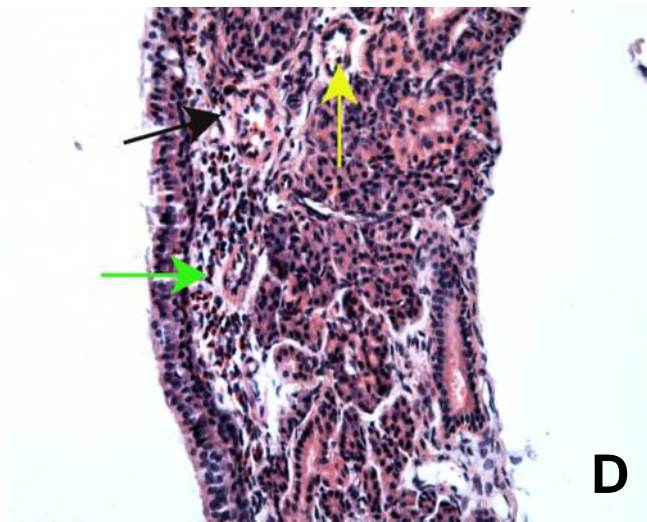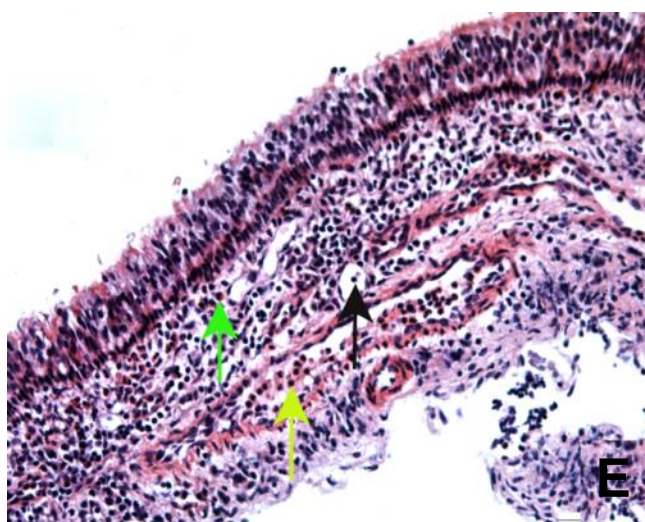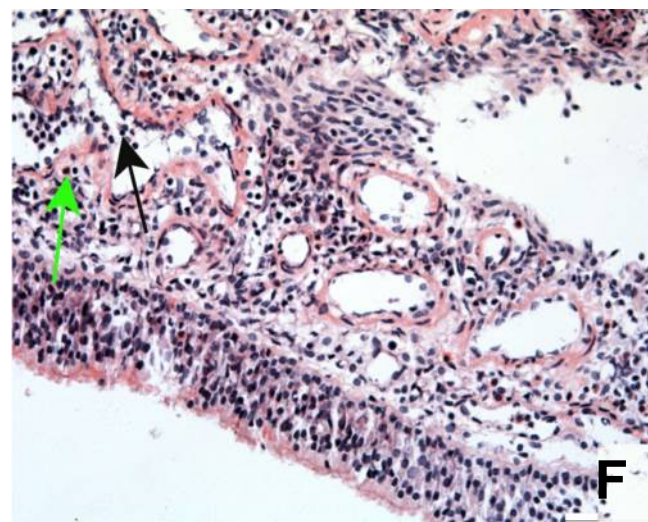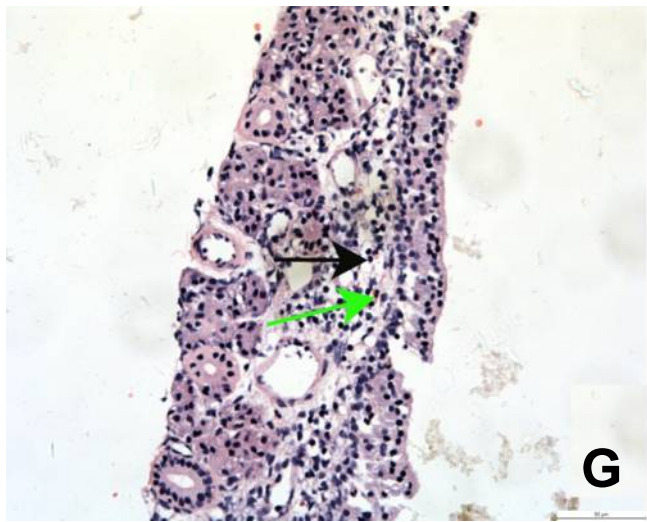

Supplement: Supplementary file 1 — Pathology of nasal mucosa inflammation: Lymphocyte infiltration was occasionally observed in the lamina propria of the nasal mucosa and the mucosal epithelial cell lamina were intact at 2 and 4 hours in the healthy guinea pigs (E-Fig. 1-2 A). However, in the AR model guinea pigs and 0.1% non-specific IgY-treated guinea pigs, a large number of eosinophils, neutrophils and lymphocytes infiltrated into the mucosal epithelial cell lamina and the lamina propria of the nasal mucosa at 2 and 4 hours. There was edema in the lamina propria of the nasal mucosa, and the epithelial cells had fallen off in the mucosal epithelial cell lamina at 2 and 4 hours in the AR model guinea pigs and 0.1% non-specific IgY-treated guinea pigs (E-Fig. 1-2 B-C). In the 0.1% anti-TNF-α-treated and 0.1% anti-IL-1β-treated guinea pigs, many eosinophils, neutrophils and lymphocytes infiltrated into the mucosal epithelial cell lamina and the lamina propria of the nasal mucosa at 2 and 4 hours. There was edema in the lamina propria of the nasal mucosa in the 0.1% anti-TNF-α-treated and 0.1% anti-IL-1β-treated guinea pigs at 2 and 4 hours. Some of the epithelial cells of the mucosal epithelial cell lamina had fallen off at 2 and 4 hours in the 0.1% anti-TNF-α-treated and 0.1% anti-IL-1β-treated guinea pigs at 2 and 4 hours. The inflammatory response was heavier in the lamina propria of the nasal mucosa at 4 than at 2 hours in the 0.1% anti-TNF-α-treated and 0.1% anti-IL-1β-treated guinea pigs (E-Fig. 1-2 D-E). In the 0.1% anti-TNF-α/IL-1β-treated and fluticasone propionate-treated guinea pigs, a small number of eosinophils, neutrophils and lymphocytes infiltrated into the mucosal epithelial cell lamina and the lamina propria of the nasal mucosa, and the mucosal epithelial cell lamina was more intact in the nasal mucosa at 2 h. The edema in the lamina propria of the nasal mucosa was heavier in the lamina propria of the nasal mucosa at 2 h in the 0.1% anti-TNF-α/IL-1β-treated guinea pigs than in the fl [file 3128182.f1.zip › E Fig.1.pdf]

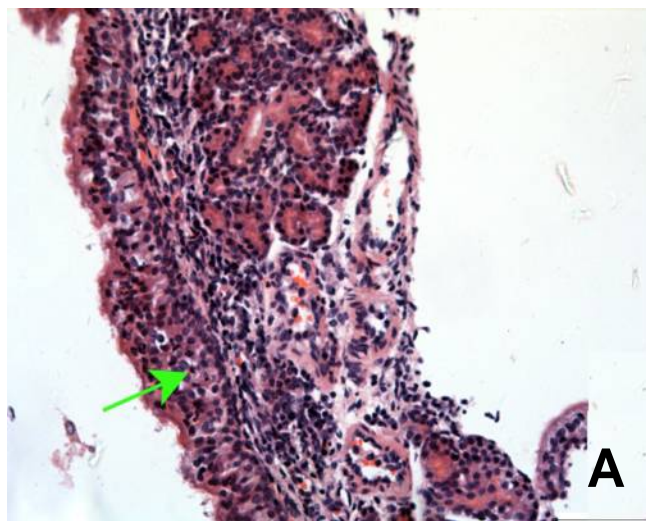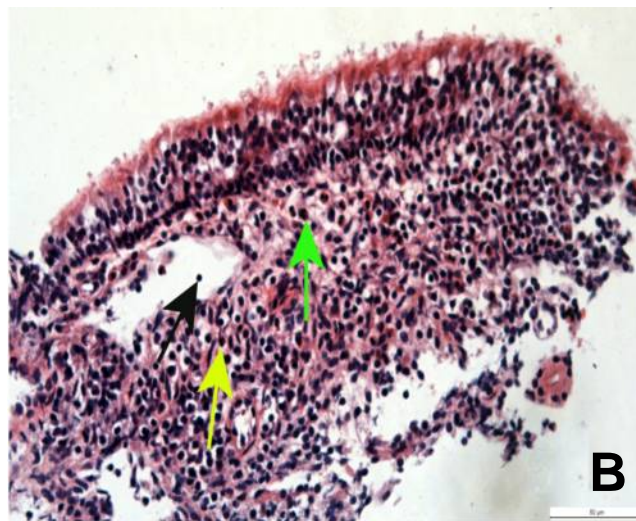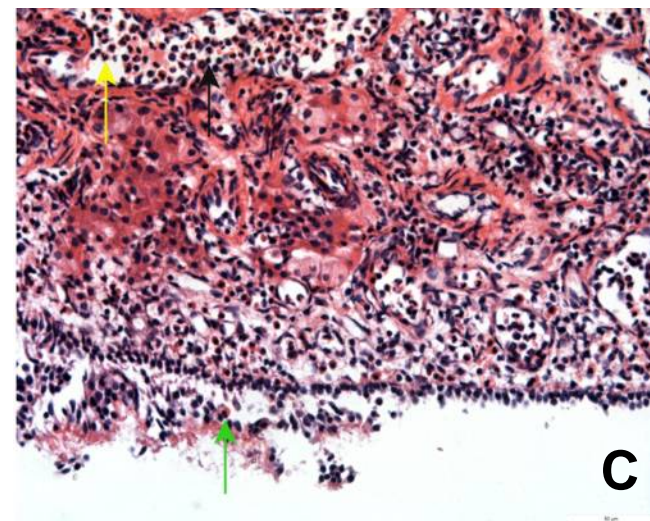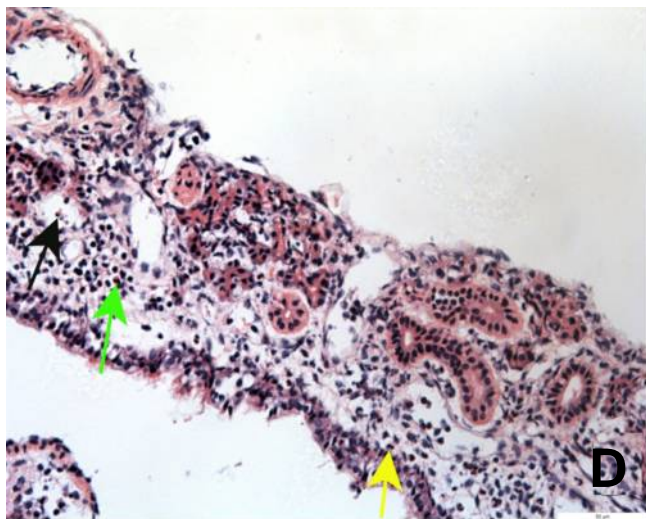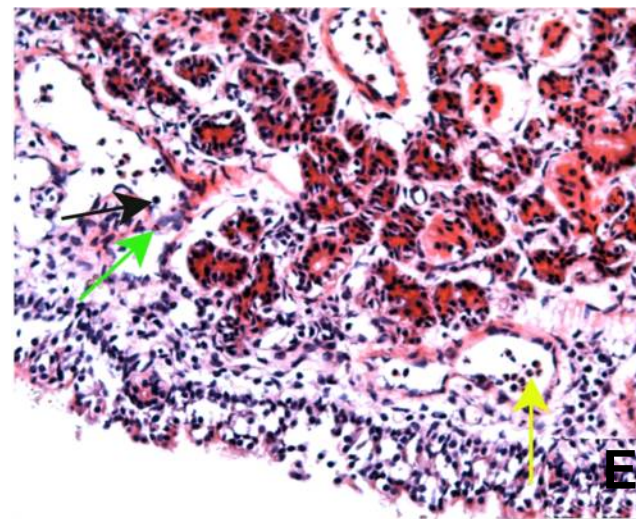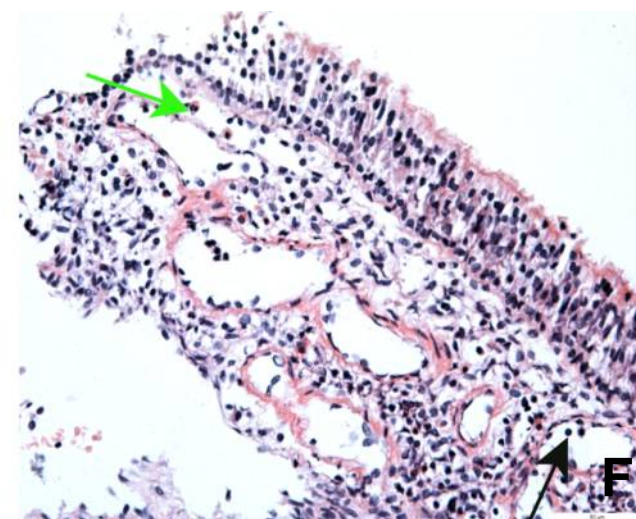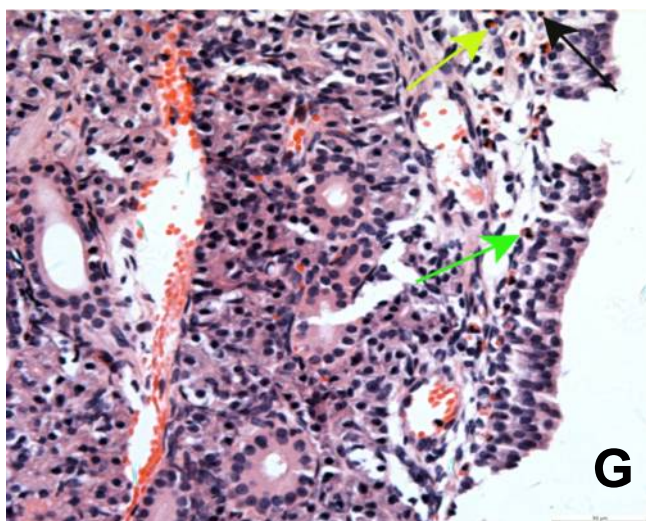

Supplement: Supplementary file 1 — Pathology of nasal mucosa inflammation: Lymphocyte infiltration was occasionally observed in the lamina propria of the nasal mucosa and the mucosal epithelial cell lamina were intact at 2 and 4 hours in the healthy guinea pigs (E-Fig. 1-2 A). However, in the AR model guinea pigs and 0.1% non-specific IgY-treated guinea pigs, a large number of eosinophils, neutrophils and lymphocytes infiltrated into the mucosal epithelial cell lamina and the lamina propria of the nasal mucosa at 2 and 4 hours. There was edema in the lamina propria of the nasal mucosa, and the epithelial cells had fallen off in the mucosal epithelial cell lamina at 2 and 4 hours in the AR model guinea pigs and 0.1% non-specific IgY-treated guinea pigs (E-Fig. 1-2 B-C). In the 0.1% anti-TNF-α-treated and 0.1% anti-IL-1β-treated guinea pigs, many eosinophils, neutrophils and lymphocytes infiltrated into the mucosal epithelial cell lamina and the lamina propria of the nasal mucosa at 2 and 4 hours. There was edema in the lamina propria of the nasal mucosa in the 0.1% anti-TNF-α-treated and 0.1% anti-IL-1β-treated guinea pigs at 2 and 4 hours. Some of the epithelial cells of the mucosal epithelial cell lamina had fallen off at 2 and 4 hours in the 0.1% anti-TNF-α-treated and 0.1% anti-IL-1β-treated guinea pigs at 2 and 4 hours. The inflammatory response was heavier in the lamina propria of the nasal mucosa at 4 than at 2 hours in the 0.1% anti-TNF-α-treated and 0.1% anti-IL-1β-treated guinea pigs (E-Fig. 1-2 D-E). In the 0.1% anti-TNF-α/IL-1β-treated and fluticasone propionate-treated guinea pigs, a small number of eosinophils, neutrophils and lymphocytes infiltrated into the mucosal epithelial cell lamina and the lamina propria of the nasal mucosa, and the mucosal epithelial cell lamina was more intact in the nasal mucosa at 2 h. The edema in the lamina propria of the nasal mucosa was heavier in the lamina propria of the nasal mucosa at 2 h in the 0.1% anti-TNF-α/IL-1β-treated guinea pigs than in the fl [file 3128182.f1.zip › E Fig.2.pdf]

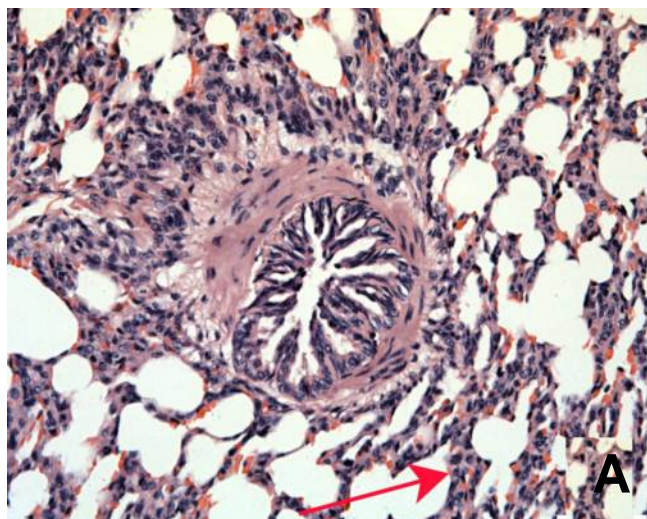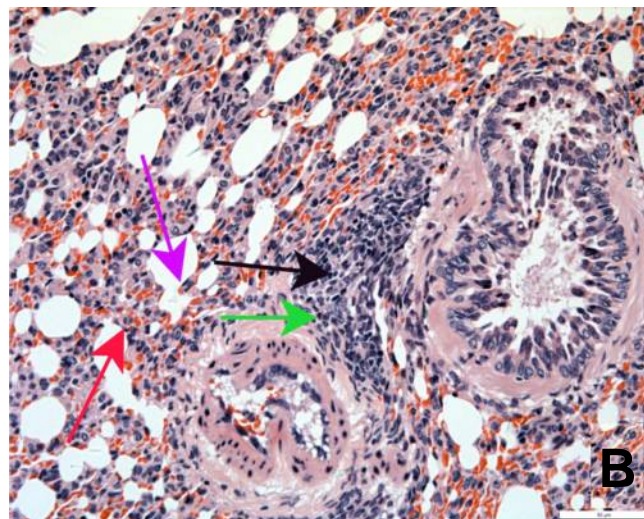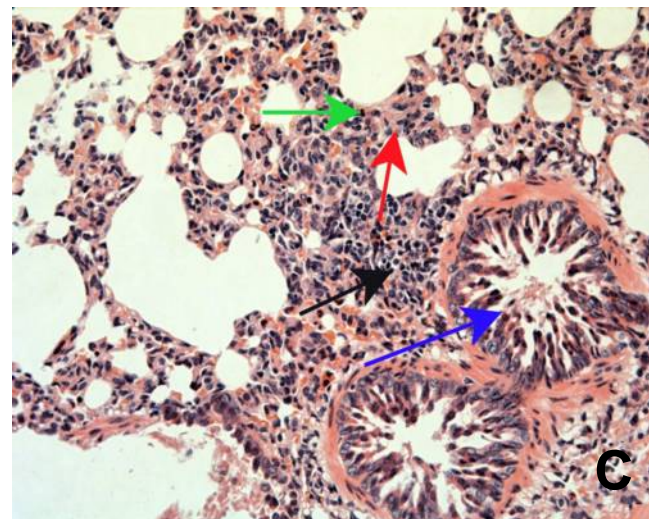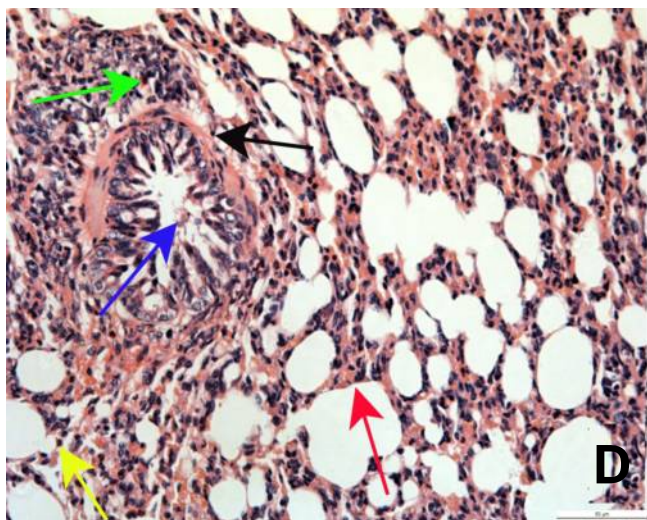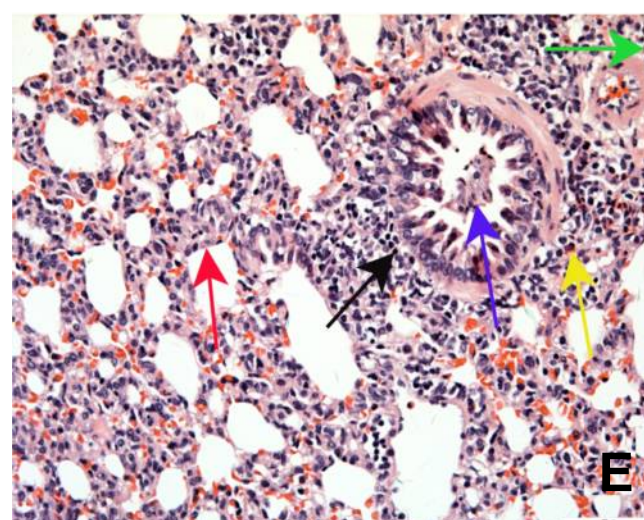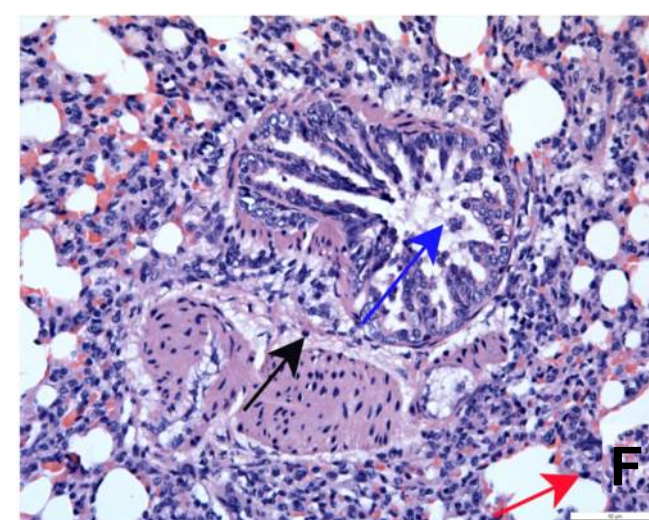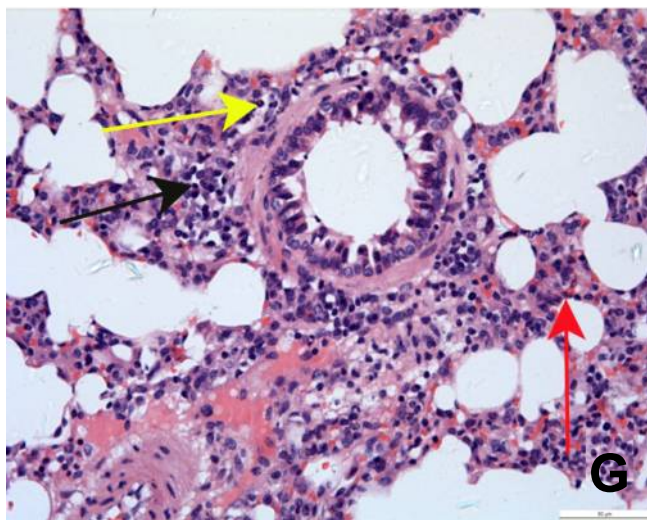

Supplement: Supplementary file 1 — Pathology of nasal mucosa inflammation: Lymphocyte infiltration was occasionally observed in the lamina propria of the nasal mucosa and the mucosal epithelial cell lamina were intact at 2 and 4 hours in the healthy guinea pigs (E-Fig. 1-2 A). However, in the AR model guinea pigs and 0.1% non-specific IgY-treated guinea pigs, a large number of eosinophils, neutrophils and lymphocytes infiltrated into the mucosal epithelial cell lamina and the lamina propria of the nasal mucosa at 2 and 4 hours. There was edema in the lamina propria of the nasal mucosa, and the epithelial cells had fallen off in the mucosal epithelial cell lamina at 2 and 4 hours in the AR model guinea pigs and 0.1% non-specific IgY-treated guinea pigs (E-Fig. 1-2 B-C). In the 0.1% anti-TNF-α-treated and 0.1% anti-IL-1β-treated guinea pigs, many eosinophils, neutrophils and lymphocytes infiltrated into the mucosal epithelial cell lamina and the lamina propria of the nasal mucosa at 2 and 4 hours. There was edema in the lamina propria of the nasal mucosa in the 0.1% anti-TNF-α-treated and 0.1% anti-IL-1β-treated guinea pigs at 2 and 4 hours. Some of the epithelial cells of the mucosal epithelial cell lamina had fallen off at 2 and 4 hours in the 0.1% anti-TNF-α-treated and 0.1% anti-IL-1β-treated guinea pigs at 2 and 4 hours. The inflammatory response was heavier in the lamina propria of the nasal mucosa at 4 than at 2 hours in the 0.1% anti-TNF-α-treated and 0.1% anti-IL-1β-treated guinea pigs (E-Fig. 1-2 D-E). In the 0.1% anti-TNF-α/IL-1β-treated and fluticasone propionate-treated guinea pigs, a small number of eosinophils, neutrophils and lymphocytes infiltrated into the mucosal epithelial cell lamina and the lamina propria of the nasal mucosa, and the mucosal epithelial cell lamina was more intact in the nasal mucosa at 2 h. The edema in the lamina propria of the nasal mucosa was heavier in the lamina propria of the nasal mucosa at 2 h in the 0.1% anti-TNF-α/IL-1β-treated guinea pigs than in the fl [file 3128182.f1.zip › E Fig.3.pdf]

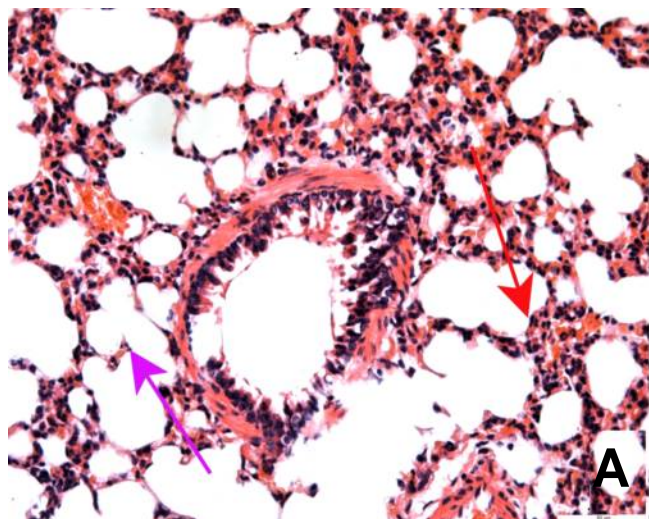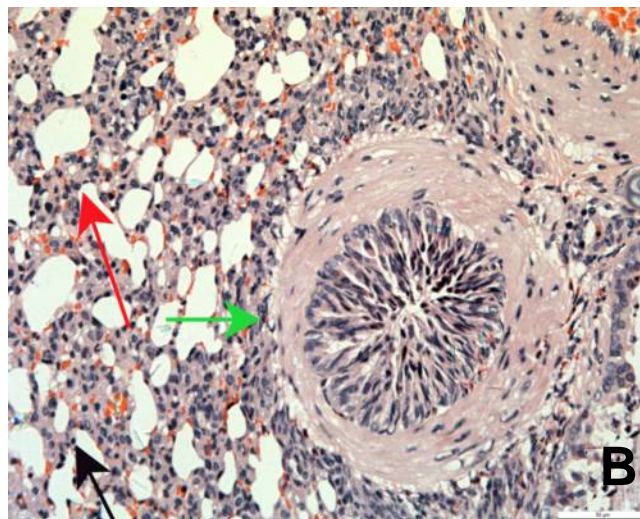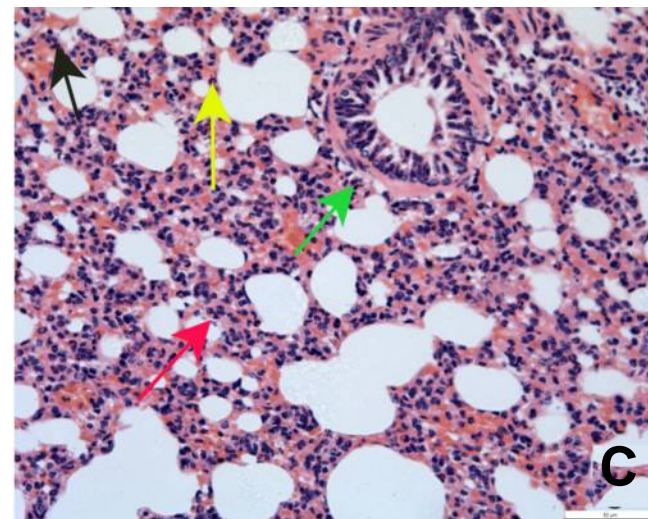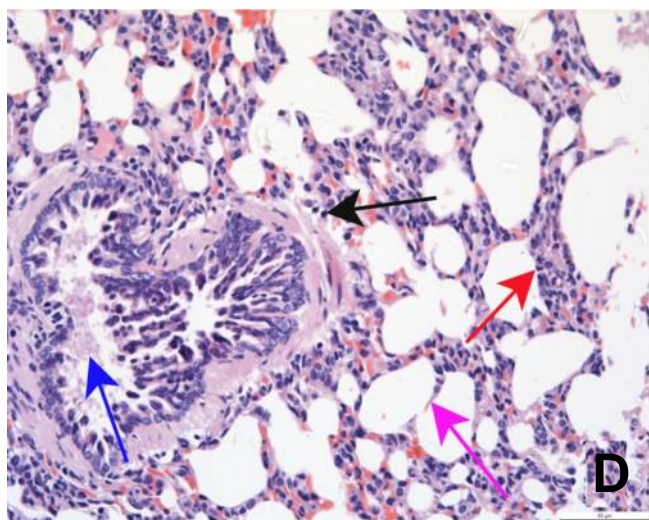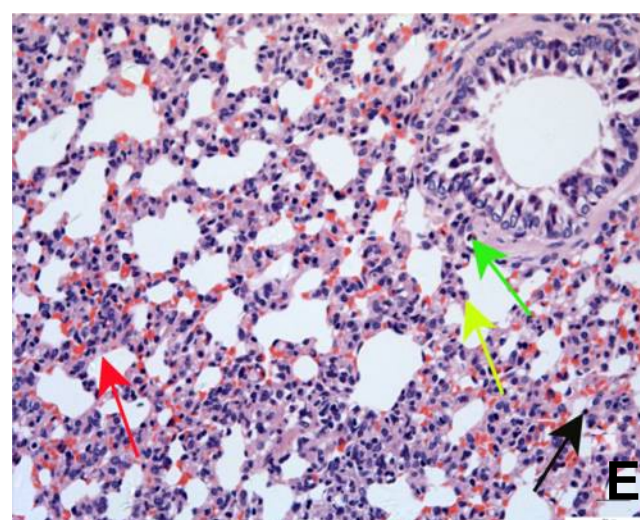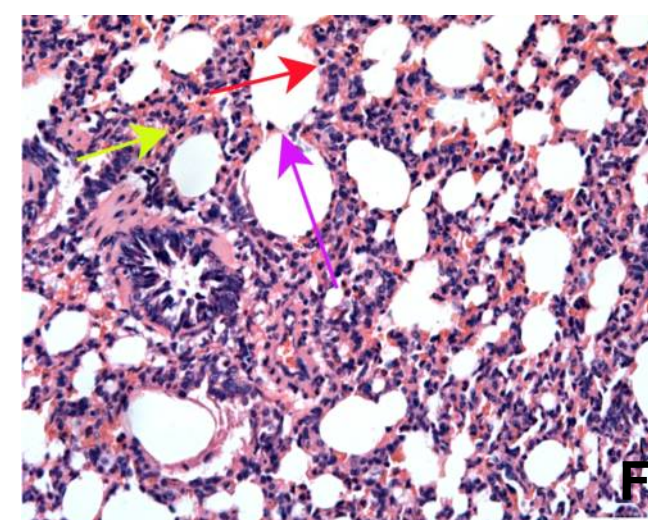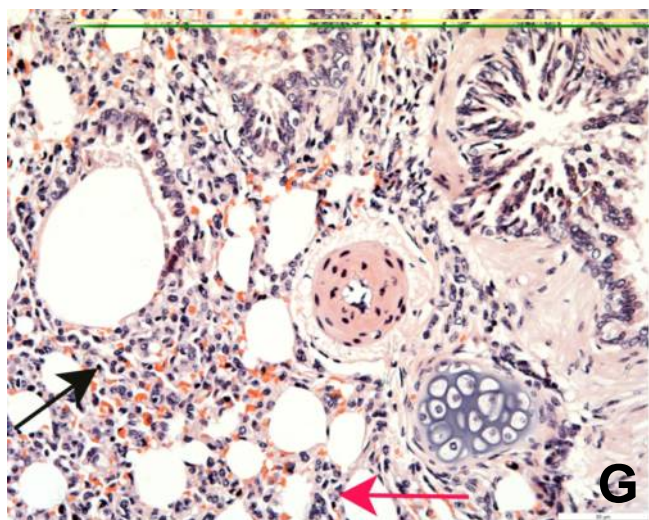

Supplement: Supplementary file 1 — Pathology of nasal mucosa inflammation: Lymphocyte infiltration was occasionally observed in the lamina propria of the nasal mucosa and the mucosal epithelial cell lamina were intact at 2 and 4 hours in the healthy guinea pigs (E-Fig. 1-2 A). However, in the AR model guinea pigs and 0.1% non-specific IgY-treated guinea pigs, a large number of eosinophils, neutrophils and lymphocytes infiltrated into the mucosal epithelial cell lamina and the lamina propria of the nasal mucosa at 2 and 4 hours. There was edema in the lamina propria of the nasal mucosa, and the epithelial cells had fallen off in the mucosal epithelial cell lamina at 2 and 4 hours in the AR model guinea pigs and 0.1% non-specific IgY-treated guinea pigs (E-Fig. 1-2 B-C). In the 0.1% anti-TNF-α-treated and 0.1% anti-IL-1β-treated guinea pigs, many eosinophils, neutrophils and lymphocytes infiltrated into the mucosal epithelial cell lamina and the lamina propria of the nasal mucosa at 2 and 4 hours. There was edema in the lamina propria of the nasal mucosa in the 0.1% anti-TNF-α-treated and 0.1% anti-IL-1β-treated guinea pigs at 2 and 4 hours. Some of the epithelial cells of the mucosal epithelial cell lamina had fallen off at 2 and 4 hours in the 0.1% anti-TNF-α-treated and 0.1% anti-IL-1β-treated guinea pigs at 2 and 4 hours. The inflammatory response was heavier in the lamina propria of the nasal mucosa at 4 than at 2 hours in the 0.1% anti-TNF-α-treated and 0.1% anti-IL-1β-treated guinea pigs (E-Fig. 1-2 D-E). In the 0.1% anti-TNF-α/IL-1β-treated and fluticasone propionate-treated guinea pigs, a small number of eosinophils, neutrophils and lymphocytes infiltrated into the mucosal epithelial cell lamina and the lamina propria of the nasal mucosa, and the mucosal epithelial cell lamina was more intact in the nasal mucosa at 2 h. The edema in the lamina propria of the nasal mucosa was heavier in the lamina propria of the nasal mucosa at 2 h in the 0.1% anti-TNF-α/IL-1β-treated guinea pigs than in the fl [file 3128182.f1.zip › E Fig.4.pdf]
