# Supplementary material for: Anti-Interleukin-1 Beta/Tumor Necrosis Factor-Alpha IgY Antibodies Reduce Pathological Allergic Responses in Guinea Pigs with Allergic Rhinitis
Source: Mediators Inflamm. 2016 Mar 7;2016:3128182. doi: 10.1155/2016/3128182 (PMC4800104; doi:10.1155/2016/3128182)
Supplement: Supplementary file 1 — Pathology of nasal mucosa inflammation: Lymphocyte infiltration was occasionally observed in the lamina propria of the nasal mucosa and the mucosal epithelial cell lamina were intact at 2 and 4 hours in the healthy guinea pigs (E-Fig. 1-2 A). However, in the AR model guinea pigs and 0.1% non-specific IgY-treated guinea pigs, a large number of eosinophils, neutrophils and lymphocytes infiltrated into the mucosal epithelial cell lamina and the lamina propria of the nasal mucosa at 2 and 4 hours. There was edema in the lamina propria of the nasal mucosa, and the epithelial cells had fallen off in the mucosal epithelial cell lamina at 2 and 4 hours in the AR model guinea pigs and 0.1% non-specific IgY-treated guinea pigs (E-Fig. 1-2 B-C). In the 0.1% anti-TNF-α-treated and 0.1% anti-IL-1β-treated guinea pigs, many eosinophils, neutrophils and lymphocytes infiltrated into the mucosal epithelial cell lamina and the lamina propria of the nasal mucosa at 2 and 4 hours. There was edema in the lamina propria of the nasal mucosa in the 0.1% anti-TNF-α-treated and 0.1% anti-IL-1β-treated guinea pigs at 2 and 4 hours. Some of the epithelial cells of the mucosal epithelial cell lamina had fallen off at 2 and 4 hours in the 0.1% anti-TNF-α-treated and 0.1% anti-IL-1β-treated guinea pigs at 2 and 4 hours. The inflammatory response was heavier in the lamina propria of the nasal mucosa at 4 than at 2 hours in the 0.1% anti-TNF-α-treated and 0.1% anti-IL-1β-treated guinea pigs (E-Fig. 1-2 D-E). In the 0.1% anti-TNF-α/IL-1β-treated and fluticasone propionate-treated guinea pigs, a small number of eosinophils, neutrophils and lymphocytes infiltrated into the mucosal epithelial cell lamina and the lamina propria of the nasal mucosa, and the mucosal epithelial cell lamina was more intact in the nasal mucosa at 2 h. The edema in the lamina propria of the nasal mucosa was heavier in the lamina propria of the nasal mucosa at 2 h in the 0.1% anti-TNF-α/IL-1β-treated guinea pigs than in the fl [file 3128182.f1.zip › Supplementary Figure Legend.doc]

**eFigure legend**

**E Fig. 1 HE staining of nasal mucosal sections at 2 h in guinea pigs of the healthy control and different treated AR groups (200×)**. **A,** the healthy control group (group C); **B,** the AR model group (group M); **C,** the 0.1% non-specific IgY treated group (group Z_1_); **D,** the 0.1% anti-TNF-α treated group (group Z_2_); **E,** the 0.1% anti-IL-1β treated group (group Z_3_); **F,** the 0.1% anti-IL-1β/TNF-α treated group (group Z_4_); **G,** the fluticasone propionate treated group (group Z_5_). **Eosinophils** infiltration (the green arrows); **Neutrophils** infiltration (the yellow arrows); **Lymphocytes** infiltration (the black arrows).

**E Fig. 2 E HE staining of nasal mucosal sections at 4 h in guinea pigs of the healthy control and different treated AR groups (200×)**. **A,** the healthy control group (group C); **B,** the AR model group (group M); **C,** the 0.1% non-specific IgY treated group (group Z_1_); **D,** the 0.1% anti-TNF-α treated group (group Z_2_); **E,** the 0.1% anti-IL-1β treated group (group Z_3_); **F,** the 0.1% anti-IL-1β/TNF-α treated group (group Z_4_); **G,** the fluticasone propionate treated group (group Z_5_); **Eosinophils** infiltration (the green arrows); **Neutrophils** infiltration (the yellow arrows); **Lymphocytes** infiltration (the black arrows).

**E Fig. 3 HE staining of lung tissue sections at 2 h in guinea pigs of the healthy control and different treated AR groups (200×)**. **A,** the healthy control group (group C); **B,** the AR model group (group M); **C,** the 0.1% non-specific IgY treated group (group Z_1_); **D,** the 0.1% anti-TNF-α treated group (group Z_2_); **E,** the 0.1% anti-IL-1β treated group (group Z_3_); **F,** the 0.1% anti-IL-1β/TNF-α treated group (group Z_4_); **G,** the fluticasone propionate treated group (group Z_5_); **Eosinophils** infiltration (the green arrows); **Neutrophils** infiltration (the yellow arrows); **Lymphocytes** infiltration (the black arrows); **Broadening** of the alveolar septa (the red arrows); **Fracture** of alveolar septa (the violet arrows); **The partial drop** of the number of bronchial mucosal epithelial cells and discharge of intrabronchial secreta (the blue arrows).

**E Fig. 4 HE staining of lung tissue sections at 4 h in guinea pigs of the healthy control and different treated AR groups (200×)**. **A,** the healthy control group (group C); **B,** the AR model group (group M); **C,** the 0.1% non-specific IgY treated group (group Z_1_); **D,** the 0.1% anti-TNF-α treated group (group Z_2_); **E,** the 0.1% anti-IL-1β treated group (group Z_3_); **F,** the 0.1% anti-IL-1β/TNF-α treated group (group Z_4_); **G,** the fluticasone propionate treated group (group Z_5_); **Eosinophils** infiltration (the green arrows); **Neutrophils** infiltration (the yellow arrows); **Lymphocytes** infiltration (the black arrows); **Broadening** of the alveolar septa (the red arrows); **Fracture** of alveolar septa (the violet arrows); **The partial drop** of the number of bronchial mucosal epithelial cells and discharge of intrabronchial secreta (the blue arrows).
